# Towards cost-effective side-chain isotope labelling of proteins expressed in human cells

**DOI:** 10.1007/s10858-024-00447-6

**Published:** 2024-08-22

**Authors:** Martina Rosati, Letizia Barbieri, Matus Hlavac, Sarah Kratzwald, Roman J. Lichtenecker, Robert Konrat, Enrico Luchinat, Lucia Banci

**Affiliations:** 1 CERM ─ Magnetic Resonance Center, Università degli Studi di Firenze, Sesto Fiorentino, Italy; 2 Consorzio Interuniversitario Risonanze Magnetiche di Metallo Proteine ─ CIRMMP, Sesto Fiorentino, Italy; 3 MAG-LAB GmbH, Vienna, Austria; 4 Institute of Organic Chemistry, University of Vienna, Vienna, Austria; 5 Department of Structural and Computational Biology, Max Perutz Laboratories, University of Vienna, Vienna, Austria; 6 Dipartimento di Chimica, Università degli Studi di Firenze, Sesto Fiorentino, Italy

**Keywords:** Protein labelling, Alpha-ketoacid, In-cell NMR, HEK293T cells, Mammalian expression, Transaminases

## Abstract

Side chain isotope labelling is a powerful tool to study protein structure and interactions by NMR spectroscopy. ^1^H,^13^C labelling of side-chain methyl groups in a deuterated background allows studying large molecules, while side-chain aromatic groups are highly sensitive to the interaction with ligands, drugs, and other proteins. In *E. coli*, side chain labelling is performed by substituting amino acids with isotope-labelled precursors. However, proteins that can only be produced in mammalian cells require expensive isotope-labelled amino acids. Here we provide a simple and cost-effective method to label side chains in mammalian cells, which exploits the reversible reaction catalyzed by endogenous transaminases to convert isotope-labelled α-ketoacid precursors. We show by in-cell and in-lysate NMR spectroscopy that replacing an amino acid in the medium with its cognate precursor is sufficient to achieve selective labelling without scrambling, and how this approach allows monitoring conformational changes such as those arising from ligand binding.

**Supplementary Information:**

The online version contains supplementary material available at 10.1007/s10858-024-00447-6.

## Introduction

Interactions underpin all biological processes. They occur between proteins, between proteins and their biological ligands and inhibitors, or between proteins and synthetic molecules. NMR spectroscopy is ideally suited to investigate such interactions at atomic resolution, in physiologically relevant settings, e.g. in intact cells and lysates (Theillet and Luchinat 2022). The various types of interactions involve specific types of amino acids. Therefore, it is essential to develop novel, efficient, and cost-effective technologies to examine interactions by looking at the side chains of the specific amino acids relevant for molecular interactions (Schörghuber et al. 2018). Furthermore, structural characterization of large proteins by NMR requires residue-specific labelling in order to improve spectral resolution and sensitivity, and to reduce signal overlap. Two side chain labelling types are particularly useful to investigate protein structure, dynamics and interactions: ^1^H,^13^C labelling of aromatic residues and methyl-^1^H,^13^C labelling of aliphatic residues. Aromatic residues are often involved in the interaction with other proteins, ligands or drugs, and take part in enzymatic activities (Serrano et al. 1991; Schörghuber et al. 2018; Ninković et al. 2020). On the other hand, labelled methyl groups of aliphatic residues are highly useful when investigating slow-tumbling macromolecules, thanks to their high intrinsic mobility and three-fold proton multiplicity, which result in a greatly enhanced sensitivity. Methyl labelling allows for the recording of NMR experiments of large proteins that would otherwise feature exceedingly broadened signals due to fast transverse relaxation (Kerfah et al. 2015). In cells, a similar scenario is also encountered when studying small globular proteins which interact with large, abundant cellular components (Barbieri et al. 2015; Burz et al. 2019). In both cases, the presence of a deuterated side chain background further increases the sensitivity by decreasing the ^1^H spin relaxation rates. Moreover, sparse labelling schemes greatly simplify the spectra of large proteins by reducing spectral overlap.

In *E. coli*, side chain labelling is typically performed by substituting amino acids with the cognate labelled α-ketoacid precursors to the growth medium, as these compounds can be synthesized from commercially available isotope sources without the need of creating chiral carbons by elaborate organic synthesis. Isotope-labelled α-ketoacids are then effectively incorporated into the host organism’s biosynthetic pathways and converted into the respective amino acids by bacterial transaminases, which substitute the keto group with an amino group in vivo (Goto et al. 1999; Lichtenecker et al. 2004, 2013a, c). Although this method is extremely effective, it has never been applied to those proteins, which require production in mammalian cells. Indeed, previous reports on selective isotope labelling in mammalian cells had to make use of synthetically more demanding and thus more expensive isotope-labelled amino acids as additives to substitute the unlabelled target residues (Werner et al. 2008; Banci et al. 2013). In mammals, transaminases reversibly catalyze the first catabolic reaction in the degradation of amino acids, transferring their amino group to a ketoacid substrate, usually α-ketoglutarate, in order to obtain the corresponding α-ketoacid and glutamate (Caligiore et al. 2022). As some transaminases are known to be expressed inside mammalian tissues (Caligiore et al. 2022; Neinast et al. 2019; Toyokawa et al. 2021; Sivaraman and Kirsch 2006; Mehere et al. 2010; Cooper 2004), we reasoned that supplementing expression media with α-ketoacids should result in the biosynthesis of the corresponding amino acids in cultured mammalian cells, thus allowing the expression of proteins with amino acid-specific labelling schemes at a lower cost.

**Scheme 1:**
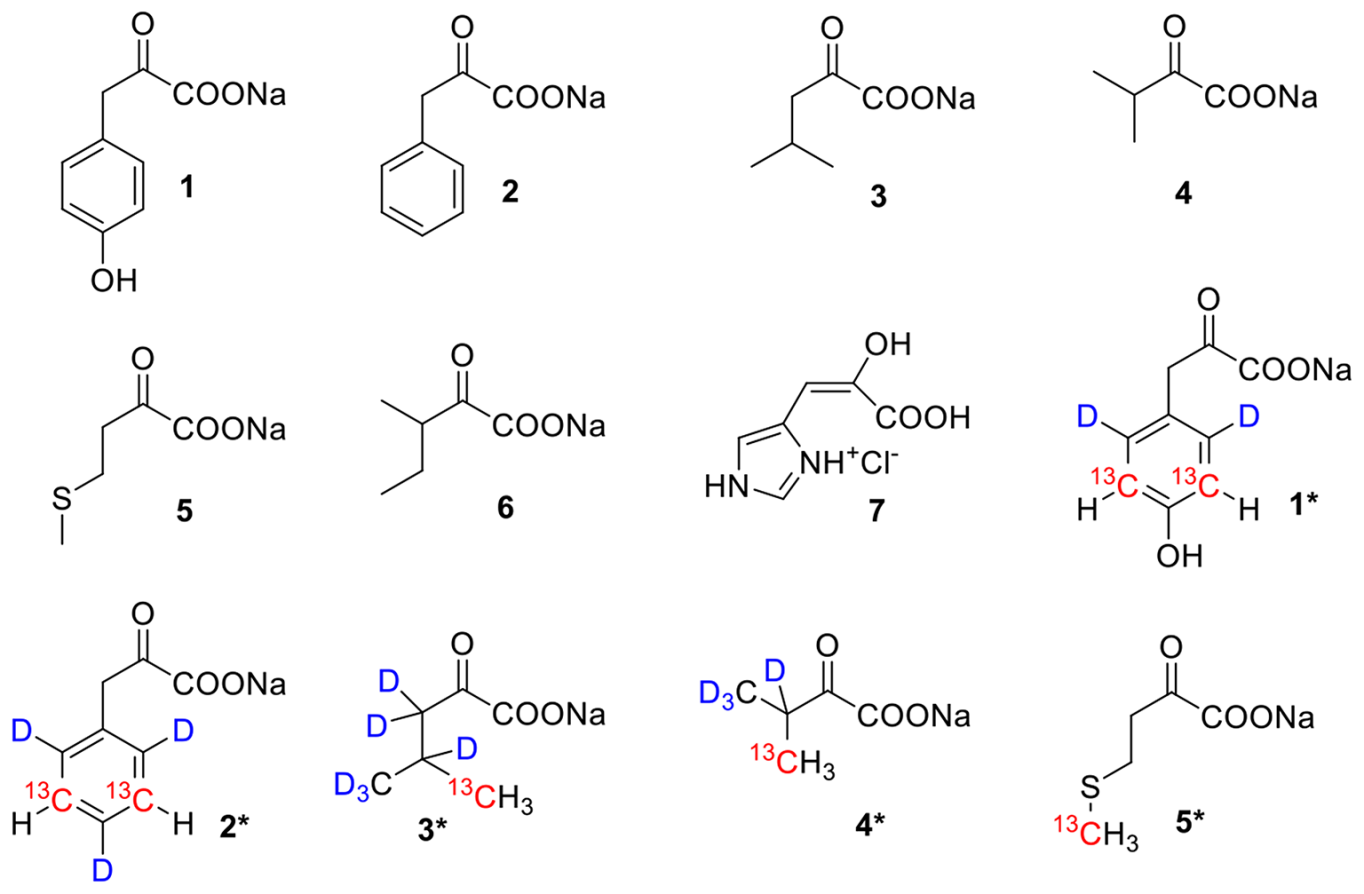
Amino acid precursors used in this study. p-Hydroxyphenylpyruvate **1**; Phenylpyruvate **2**; α-Ketoisocaproate **3**; α-Ketoisovalerate **4**; 4-Methylthio-α-ketobutanoate **5**; 3-Methyl-α-ketovalerate **6**; 4-(2-Carboxy-2-hydroxyvinyl)-*1 H*-imidazolium chloride **7**; ([3,5-^13^C_2_, 2,6-^2^H_2_] p-Hydroxyphenyl)pyruvate 1*; ([3,5-^13^C_2_, 2,4,6-^2^H_3_] Phenyl)pyruvate 2*; [5-^13^ C, 3,3,4,5,5,5-^2^H_6_] α-Ketoisocaproate 3*; [4-^13^ C, 3, 4,4,4-^2^H_4_] α-Ketoisovalerate 4*; 4-[^13^C] Methylthio-α-ketobutanoate 5*

In this work, we show that HEK293T cells supplied with custom-made culture media containing an α-ketoacid precursor in place of the corresponding amino acid result in a high-yield transient expression of the desired proteins. These experiments were performed with the unlabelled α-ketoacid precursors of tyrosine (p-hydroxyphenylpyruvate **1**), phenylalanine (phenylpyruvate **2**), leucine (α-ketoisocaproate **3**), valine (α-ketoisovalerate **4**), methionine (4-methylthio-α-ketobutanoic acid **5**), isoleucine (3-methyl-α-ketovalerate **6**) and histidine (4-(2-carboxy-2-hydroxyvinyl)-*1H*-imidazolium chloride **7**). Selective side-chain labelling protocols were demonstrated on two model proteins, carbonic anhydrase II (CA II) and the Parkinson-related protein deglycase 1 (DJ-1), by recording fast 2D ^1^H,^13^C NMR spectra of both intact cells and cell lysates. The unlabelled precursors, as well as the corresponding heavy isotope containing isotopologues (compounds 1*-5*) are shown in scheme 1 and have been synthesized using previously reported protocols (Lichtenecker et al. 2004, 2013a; Fischer et al. 2007; Lichtenecker 2014).

## Materials and methods

### Plasmid design

Following the procedures outlined in previous studies, DNA sequences encoding for carbonic anhydrase 2 (CA II, NP_000058.1), deglycase protein 1 (DJ-1, NP_009193.2), and empty vector (pHL-empty) were cloned in the pHLsec vector, deprived of the secretion sequence (Aricescu et al. 2006).

### Custom medium preparation

The composition of the custom-made medium was made accordingly to that of commercial high-glucose Dulbecco’s modified Eagle’s medium (DMEM, Sigma D6546), excluding the amino acid(s) to be labelled or to be replaced with precursors. The components were weighed on an analytical scale and dissolved in ultrapure water. The solution was left stirring overnight at 4 °C and the pH was brought to 7.4. The medium was then filtered under a laminar flow hood with a 0.22 nm filter and stored at -80 °C in 50 mL aliquots. The labelled amino acids or precursors were supplemented to the medium from concentrated stock solutions at the time of transfection.

### Expression tests

HEK293T cells (ATCC CRL3216) were cultured in T25 flasks with DMEM (Life Technologies). At 90-95% confluence the cells were transiently transfected with the vectors in a custom-made medium where one amino acid was substituted with the corresponding precursor (MAG-LAB) in different concentrations with respect to the concentration of the amino acid (aa: precursor 1:1, 1:2, 1:5). The transfection was carried out following a previously reported protocol (Barbieri et al. 2016), in which vector and polyethylenimine (PEI, Sigma-Aldrich) were mixed in a 1:2 ratio (8.3 µg of DNA: 16.7 µg of PEI). After transfection the cells were incubated for 48 h at 37 °C, 5% CO_2_, with custom-made DMEM supplemented with 2% (v/v) fetal bovine serum (FBS, Life Technologies) and 100 µg/mL penicillin − streptomycin (Life Technologies), respectively. It was previously shown that CA II and DJ-1 after 48 h of transient expression are correctly folded and do not undergo degradation (Barbieri et al. 2018; Luchinat et al. 2020a). The cells were then harvested in 150 µL of phosphate buffered saline (PBS, Life Technologies), lysed by freeze and thaw and the soluble part of the lysate was collected after centrifugation. The expression level of CA II was evaluated by Coomassie-stained SDS-polyacrylamide gel electrophoresis (SDS-PAGE). The loading sample, consisting in 7 µL of a solution made by diluting the soluble lysate 1:3 in 100mM Tris-HCl pH 6.8, 12.5% (v/v) glycerol, 2% (w/v) sodium dodecyl sulfate (SDS), 0.1% (w/v) bromophenol blue, and 50 mM dithiothreitol (DTT), was loaded onto pre-cast gels (Bio-Rad) and run at 200 V for 30 min. The gels were then stained with Coomassie Blue dye (ProBlue Safe Stain, Giotto Biotech).

### Protein quantification

CA II concentration in the lysates from each condition was quantified by densitometric analysis on Coomassie-stained SDS-PAGE. Three dilutions of the lysates of the expression tests of CA II with two doses of each precursor, and with commercial DMEM were loaded onto a pre-cast gel as previously described and compared to a linear regression standard curve made of four samples of purified CA II at increasing concentrations (1, 2, 5, 10 µM). The gel was stained with Coomassie Blue dye, imaged by ChemiDoc XRS (Bio-Rad), and analyzed with Image J. Densitometry was used to quantify the protein expression based on the standard curve.

### NMR sample preparation

HEK293T cells cultured in T75 flasks with DMEM were transiently transfected in custom-made medium with one amino acid substituted by a 2x dose of the cognate labelled precursor. Cell transfection was performed following the aforementioned protocol scaled up for T75 flasks (25 µg of DNA: 50 µg of PEI). Protein expression was carried out for 48 h in custom-made DMEM as above. Cells were harvested by trypsinization and suspended in 180 µL of NMR buffer (DMEM, 70 mM HEPES, and 20% (v/v) D_2_O), transferred in a 3 mm Shigemi NMR tube, gently pelleted and inserted in the NMR instrument for analysis. At the end of the NMR experiment the cells were collected and lysed in PBS buffer by freeze and thaw as described before. The soluble part of the lysate was supplemented with 10% D_2_O, transferred in a 3 mm NMR tube and inserted in the NMR instrument for analysis. For samples supplied with two precursors at the same time, the experiment was carried out with the same protocol using a custom-made medium containing both precursors and lacking the corresponding amino acids.

### NMR spectra acquisition and analysis

All NMR spectra were recorded at 310 K on a 950 MHz ^1^H Avance III (Bruker) with pulse sequences, acquisition windows and parameters specifically selected for each labelled residue (Table 1). Aromatic-labelled samples were analyzed with 2D ^1^H-^13^C SOFAST HMQC spectra (Schanda and Brutscher 2005; Sathyamoorthy et al. 2014), while methyl-labelled samples were analyzed with either 2D ^1^H-^13^C ALSOFAST or 2D ^1^H-^13^C XL-ALSOFAST HMQC spectra (Mueller 2008; Rößler et al. 2020). The experiments were performed with the same spectral window for both in-cell and lysate samples; for lysates we increased the size and decreased the number of scans per increment to increase the FID resolution while keeping constant the duration of the experiment (Table 1). Peak counting was performed automatically in Topspin and manually edited to account for partial peak overlap and to exclude peaks arising from the cellular background. CA II concentration in the cell lysates analyzed by NMR was estimated by comparing the signal intensity in the imino region of 1D ^1^H NMR spectra, which is free from cellular background signals, with those of a reference cell lysate sample containing CA II recorded with identical acquisition parameters. The concentration of CA II in the reference lysate sample (240 µM) was determined by titration with the CA inhibitor methazolamide (MZA), previously shown to bind CA II in the slow exchange regime (Luchinat et al. 2020a).

### CA II-ligand interaction

CA II was overexpressed in presence of compound 4* and treated with inhibitors to validate the labelling method for the study of interactions. After transfection and incubation for 48 h the cells were treated with one of the following inhibitors: acetazolamide (AAZ) 50 μM for 2 h, MZA 10 μM for 1 h, ethoxzolamide (ETZ) 10 μM for 1 h. After treatment, cells were collected and the samples were prepared and analyzed as reported above.

**Table 1:** NMR acquisition parameters

Precursor		1*	2*	3*	4*	5*	1*+2*	3*+4*
**Pulse sequence**		SOFAST HMQC	SOFAST HMQC	XL ALSOFAST HMQC	ALSOFAST HMQC	XL ALSOFAST HMQC	SOFAST HMQC	XL ALSOFAST HMQC
**Duration (minutes)**		44	44	61	46	59	44	61
^**1**^ **H spectral width (ppm)**		17.5	17.5	13	16	13	17.5	13
^**13**^ **C spectral width (ppm)**		13	13	30	25	20	16	30
^**13**^ **C frequency offset (ppm)**		117.6	130.6	25.1	22.6	17.6	124.6	25.1
^**13**^ **C FID size**	**in-cells**	64	64	128	128	128	80	152
	**lysate**	96	96	256	256	256	116	256
**Dummy scans**		64	64	64	64	64	64	64
**Scans**	**in-cells**	96	96	64	64	64	80	56
	**lysate**	64	64	32	32	32	56	32
**Interscan delay (ms)**		350	350	350	250	350	350	350

## Results

### Protein expression with α-ketoacid precursors

Custom-made media were prepared, in which a single α-ketoacid precursor was supplemented in place of the corresponding amino acid. Tyrosine precursor **1**, Phenylalanine precursor **2**, Leucine precursor **3**, Valine precursor **4**, Methionine precursor **5**, Isoleucine precursor **6** and Histidine precursor **7** were tested (Scheme 1). For subsequent NMR analysis, isotope-labelled precursors were employed (1*-5*, Scheme 1 and Table 2).

**Table 2:** Labelled precursors employed in this study

Precursor	Extended name	Labelling scheme
1*	[^13^C_2_, ^2^H_2_] Hydroxyphenylpyruvate	Aromatic ^13^C-^1^H, deuterated ring
2*	[^13^C_2_, ^2^H_3_] Phenylpyruvate	Aromatic ^13^C-^1^H, deuterated ring
3*	[^13^C, ^2^H_6_] α-Ketoisocaproate	Methyl ^13^CH_3_, deuterated side chain
4*	[^13^C, ^2^H_4_] α-Ketoisovalerate	Methyl ^13^CH_3_, deuterated side chain
5*	[^13^C] Methylthio- α-ketobutanoate	Methyl ^13^CH_3_

**Fig. 1:**
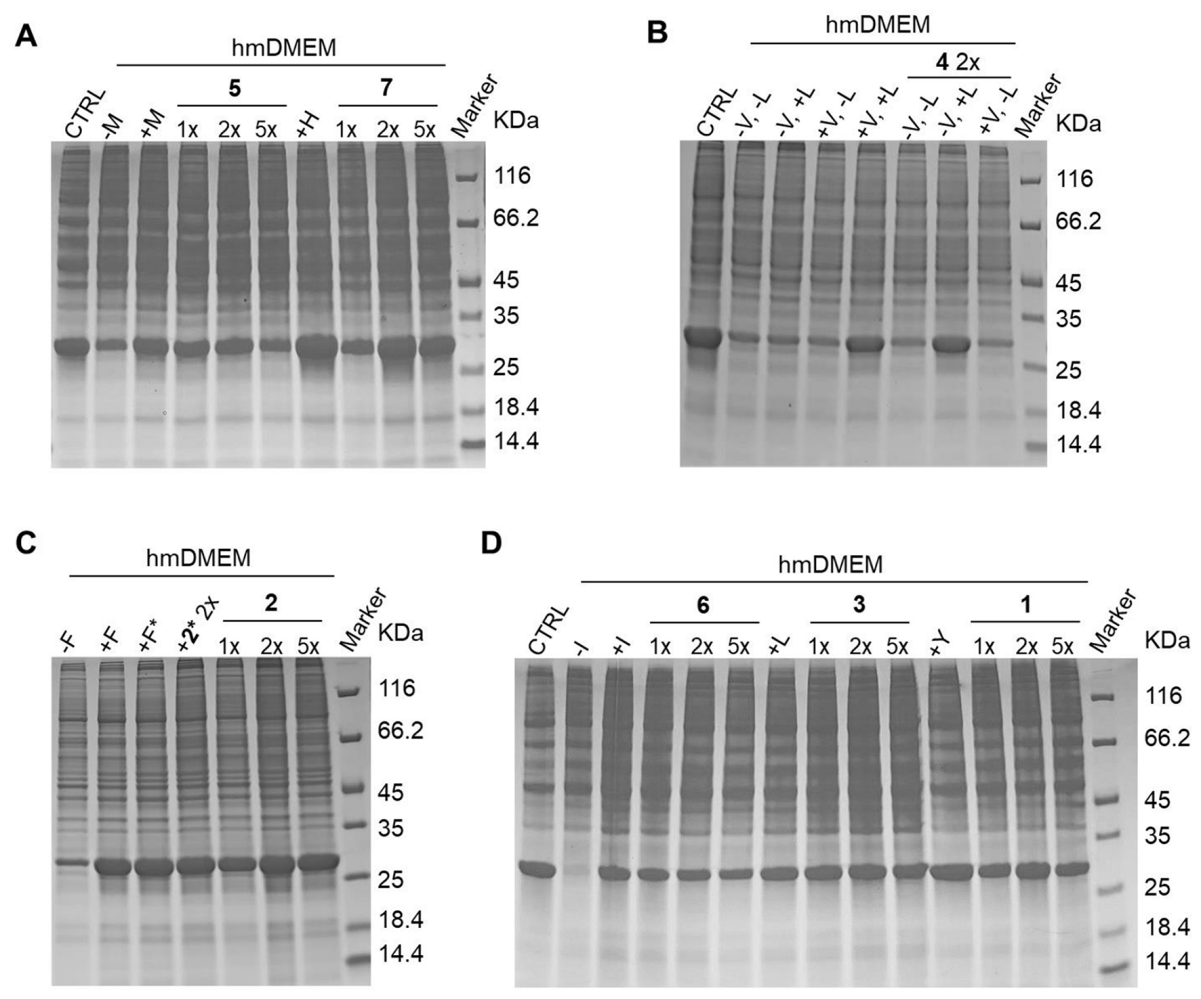
Protein expression tests. **(A**) HEK293T overexpressing CAII, incubated with commercial DMEM, custom-made DMEM w/o Met, custom-made complete DMEM, custom-made DMEM w/ Methionine precursor **5** 1x, custom-made DMEM w/ **5** 2x, custom-made DMEM w/ **5** 5x, custom-made complete DMEM, custom-made DMEM w/ Histidine precursor **7** 1x, custom-made DMEM w/ **7** 2x, custom-made DMEM w/ **7** 5x, protein marker. (**B**) HEK293T overexpressing CAII, incubated with commercial DMEM, custom-made DMEM w/o Val and Leu, custom-made DMEM w/o Val, custom-made DMEM w/o Leu, custom-made complete DMEM, custom-made DMEM w/ Valine precursor **4** 2x w/o Val and Leu, custom-made DMEM w/ **4** 2x w/o Val, custom-made DMEM w/ **4** 2x w/o Leu, protein marker. (**C**) HEK293T overexpressing CAII, incubated with custom-made DMEM w/o Phe, custom-made complete DMEM, custom-made DMEM w/ Phenylalanine precursor **2**, custom-made DMEM w/ labelled Phenylalanine precursor 2*, custom-made DMEM w/ **2** 1x, custom-made DMEM w/ **2** 2x, custom-made DMEM w/ **2** 5x, protein marker. (**D**) HEK293T overexpressing CAII, incubated with commercial DMEM, custom-made DMEM w/o Ile, custom-made complete DMEM, custom-made DMEM w/ Isoleucine precursor **6** 1x, custom-made DMEM w/ **6** 2x, custom-made DMEM w/ **6** 5x, custom-made complete DMEM, custom-made DMEM w/ Tyrosine precursor **1** 1x, custom-made DMEM w/ **1** 2x, custom-made DMEM w/ **1** 5x, protein marker

**Fig. 2:**
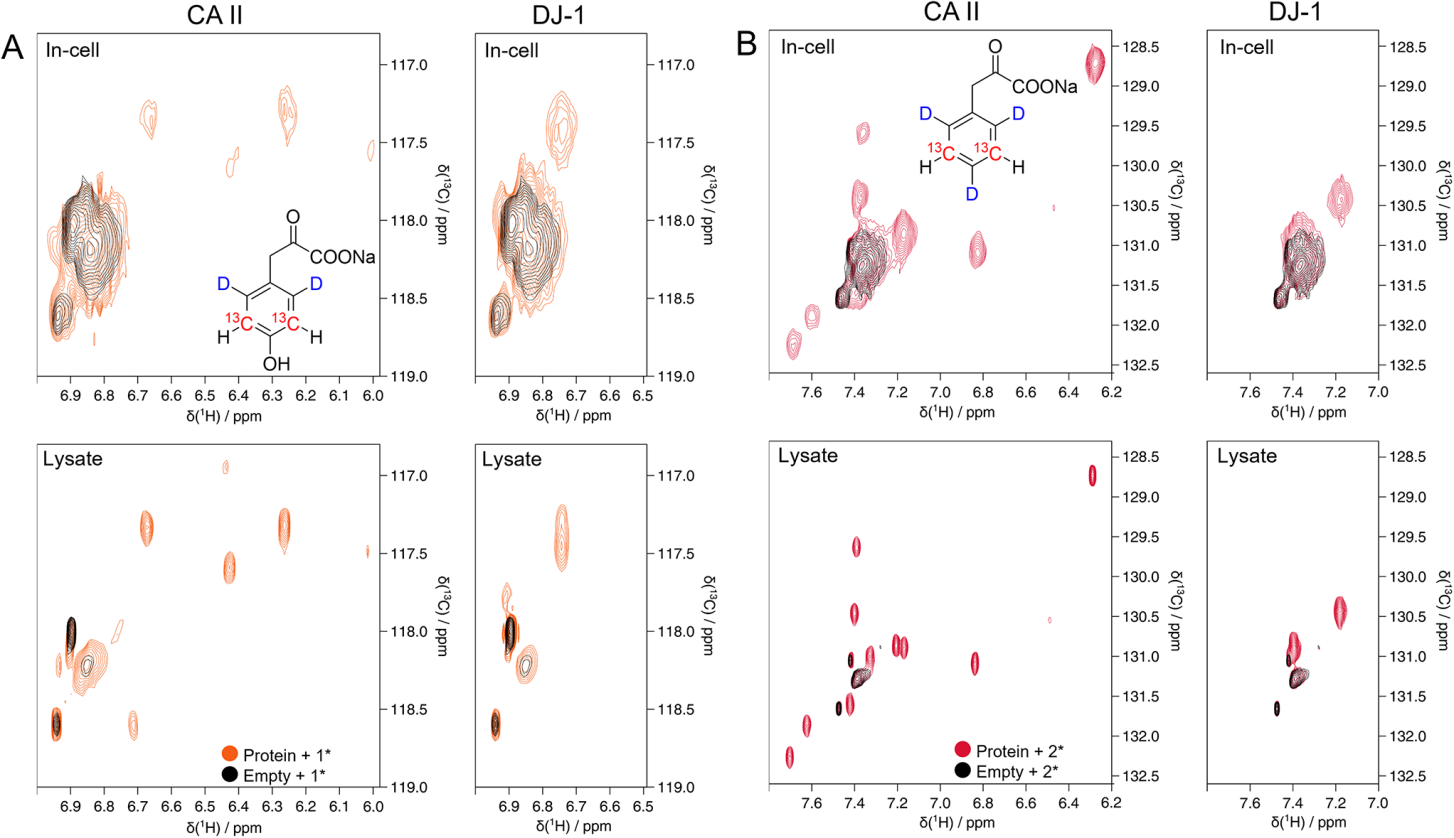
NMR spectra of tyrosine and phenylalanine-labelled proteins. **(A**) Top: In-cell spectra on HEK293T cells overexpressing CA II (left) and DJ-1 (right), incubated with 1* (orange); HEK293T cells transfected with empty vector, incubated with 1* (black). Bottom: In-solution spectra on HEK293T lysate. Lysate of cells overexpressing CA II (~ 20 µM, left) and DJ-1 (right), incubated with 1* (orange); lysate of cells transfected with empty vector, incubated with 1* (black). (**B**) Top: In-cell NMR spectra on HEK293T cells overexpressing CA II (left) and DJ-1 (right), incubated with 2* (red); HEK293T cells transfected with empty vector, incubated with 2* (black). Bottom: NMR spectra on HEK293T lysate. Lysate of cells overexpressing CA II (~ 40 µM, left) and DJ-1 (right), incubated with 2* (red); lysate of cells transfected with empty vector, incubated with 2* (black)

Protein expression efficiency in media supplemented with each precursor was tested by evaluating the expression level of CA II by SDS-PAGE. Each expression test was carried out with increasing amount of precursor (1x, 2x, and 5x doses relative to the concentration of the corresponding amino acid in the commercial DMEM) and compared to the expression in commercial DMEM (Fig. 2). For all precursors tested, the 2x dose was sufficient to achieve expression levels comparable to those obtained with the corresponding amino acid. Lower levels were obtained with both 1x dose and 5x dose of precursor, the latter being most likely a result of cytotoxic effects due to the high concentration of precursor (Fig. 1). Quantitative analysis revealed that in the lysates from cells treated with DMEM (either commercial or custom-made) CA II concentration was ~ 100 µM, whereas with 1x, 2x and 5x doses of precursor CA II was ~ 35–70 µM, 70–100 µM and 45–85 µM, respectively (Fig. S1).

In *E. coli*, α-kesoisovalerate **4**, acts as a precursor for both valine and leucine (Lichtenecker et al. 2013b). However, the pathway for the synthesis of leucine from α-ketoisovalerate is known to be inactive in mammalian cells (Neinast et al. 2019). Nevertheless, protein expression was tested in media lacking only valine, only leucine, or both. As expected, expression was only achieved in the medium lacking valine, confirming that α-ketoisovalerate only works as a valine precursor in mammalian cells (Fig. 1B).

### Incorporation of isotopically labelled precursors

For a subset of the above precursors (Table 2), protein side chain-specific isotopic labelling was evaluated through NMR spectroscopy in both intact cells and cell lysates. To this aim, two model globular proteins, CA II and DJ-1, were transiently overexpressed in presence of isotope-labelled precursors at the optimal doses reported above. To correctly identify the signals arising from the isotope-labelled overexpressed proteins, the spectra were compared with those obtained from control cells transfected with an empty vector and incubated in the same labelled media.

### Aromatic labelling

We tested the incorporation of tyrosine and phenylalanine precursors 1* and 2* via in-cell and in-lysate NMR (Fig. 2) and compared the spectra of proteins obtained from a precursor-containing medium to those obtained from a medium containing the corresponding side-chain labelled amino acids, which have been synthetized according to literature (Young et al. 2021) (Supp. Fig. S2). The ensuing in-cell NMR spectra revealed well-defined peaks for the side chain-labelled amino acids. The same peaks were better resolved in the lysate NMR spectra. Peak counting revealed a lower number of peaks than expected (Table 3). Notably, however, the same number of signals was also observed in the spectra obtained using the cognate amino acids (Supp. Fig. S2), indicating that the missing peaks do not arise from incomplete incorporation, but are likely caused by signal overlap and/or exchange broadening. Taken together, these results confirm that the α-ketoacids are transformed in the corresponding amino acids and subsequently integrated in the protein sequence at the correct positions.

### Methyl labelling

**Fig. 3:**
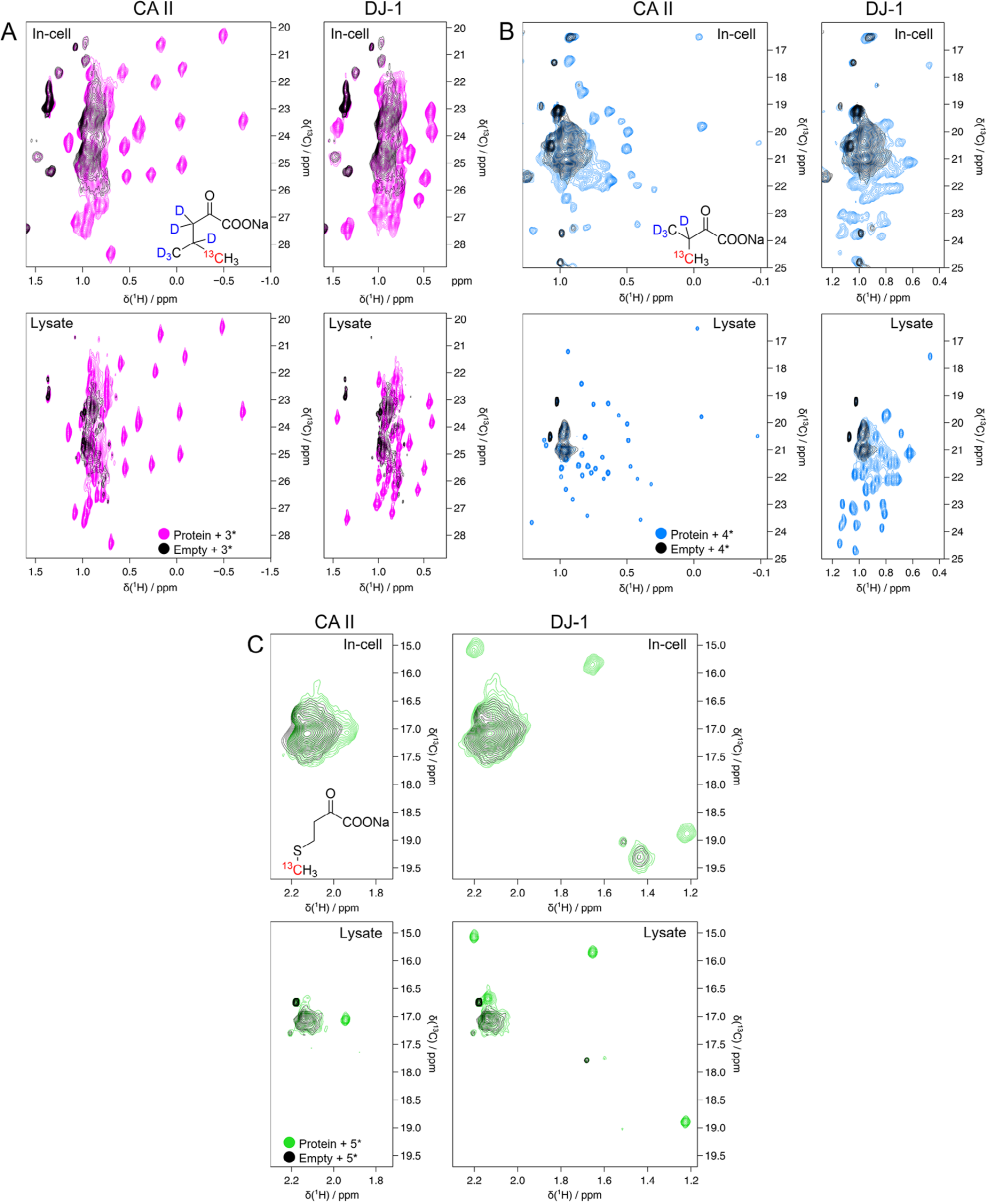
NMR spectra of valine, leucine and methionine-labelled proteins. **(A**) Top: In-cell NMR spectra on HEK293T cells overexpressing CA II (left) and DJ-1 (right), incubated with 3* (magenta); HEK293T cells overexpressing empty vector, incubated with 3* (black). Bottom: NMR spectra on HEK293T lysate. Lysate of cells overexpressing CA II (~ 50 µM, left) and DJ-1 (right), incubated with 3* (magenta); lysate of cells transfected with empty vector, incubated with 3* (black). (**B**) Top: In-cell NMR spectra on HEK293T cells overexpressing CA II (left) and DJ-1 (right), incubated with 4* (light blue); HEK293T cells transfected with empty vector, incubated with 4* (black). Bottom: NMR spectra on HEK293T lysate. Lysate of cells overexpressing CA II (~ 25 µM, left) and DJ-1 (right), incubated with 4* (light blue); lysate of cells transfected with empty vector, incubated with 4* (black). (**C**) Top: In-cell NMR spectra on HEK293T cells overexpressing CA II (left) and DJ-1 (right), incubated with 5* (green); HEK293T cells transfected with empty vector, incubated with 5* (black). Bottom: NMR spectra on HEK293T lysate. Lysate of cells overexpressing CA II (~ 25 µM, left) and DJ-1 (right), incubated with 5* (green); lysate of cells transfected with empty vector, incubated with 5* (black)

We performed in-cell and in-lysate NMR experiments on protein samples methyl-^13^C labelled at valine, leucine, and methionine residues using compounds 3*, 4* and 5*. The resulting in-cell NMR spectra showed well-resolved peaks corresponding to the side chain-labelled amino acids, which were even better resolved in the lysate NMR spectra, confirming the incorporation in the protein sequence (Fig. 3). In the case of valine and leucine precursors, where the methyl groups are not stereospecifically labelled (resulting in a racemic mixture), the number of expected peaks corresponds to twice as many residues in the protein sequence, due to the labelling of both prochiral methyl groups (Fig. 3A, B). Despite the relatively low protein concentrations, ranging between 25 and 50 µM (Supp. Fig. S3), most of the expected methyl peaks were observed in the lysate spectra (Table 3), thanks to the high sensitivity and spectral resolution attained by selective methyl labelling. To assess the contribution of natural abundance ^13^C in the spectra of selectively labelled samples, a lysate from cells expressing CA II in unlabelled medium was compared to a lysate containing leucine-labelled CA II. No signals from unlabelled protein were detected within the duration of the experiment, indicating that ^13^C natural abundance does not interfere with the detection of methyl-labelled proteins (Supp. Fig. S4).

### Combined precursor incorporation

**Fig. 4:**
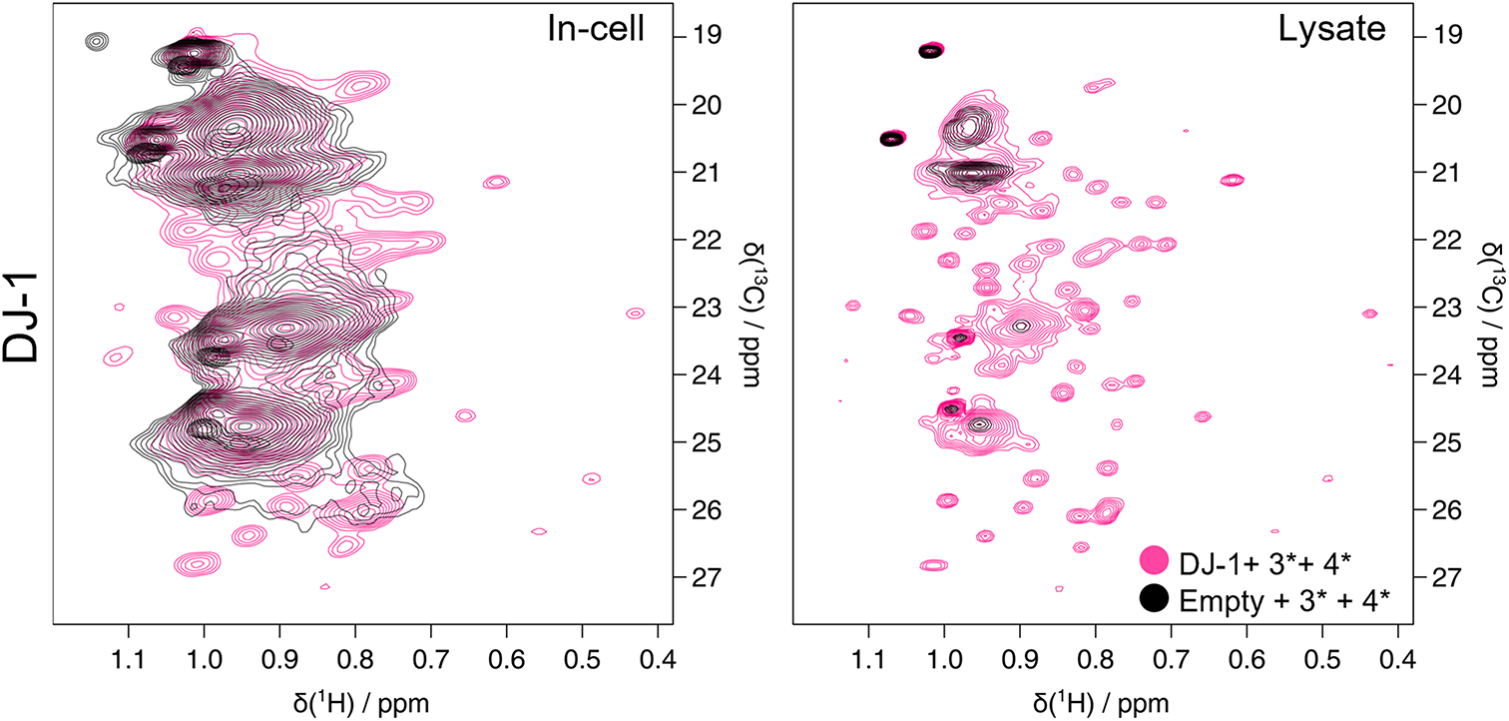
Simultaneous labelling with valine and leucine. Left: In-cell spectra on HEK293T overexpressing DJ-1, incubated with 3* and 4* (magenta); HEK293T cells overexpressing empty vector, incubated with 3* and 4* (black). Right: In-solution spectra on HEK293T lysate overexpressing DJ-1, incubated with 3* and 4* (magenta); lysate of cells overexpressing empty vector, incubated with 3* and 4* (black)

We then combined multiple precursors in the same culture medium to assess whether the expression and incorporation levels remained unchanged. We chose valine and leucine precursors, which share the same transaminase enzyme, the branched-chain amino acid aminotransferase (BCAT) (Neinast et al. 2019; Toyokawa et al. 2021). This test would thus provide further information on enzymatic overload. Therefore, a custom culture medium containing Val and Leu precursor was made and the NMR experiments was performed overexpressing DJ-1. The resulting in-cell and lysate spectra show a good DJ-1 expression level, similar to the one of DJ-1 with the precursors used separately, and an efficient incorporation of both labelled amino acids (Fig. 4).

**Table 3:** Observed vs. expected peaks detected in the NMR spectra of cell lysates. ^a^ Excluding N-terminal methionine

Precursor	Corresponding amino acid	Observed/expected peaks	
		CA II	DJ-1
1*	Tyr	5/8	3/3
2*	Phe	10/12	2/3
3*	Leu	50/52	35/36
4*	Val	34/34	36/38
5*	Met	1/1^a^	4/4^a^

### Application of the method

**Fig. 5:**
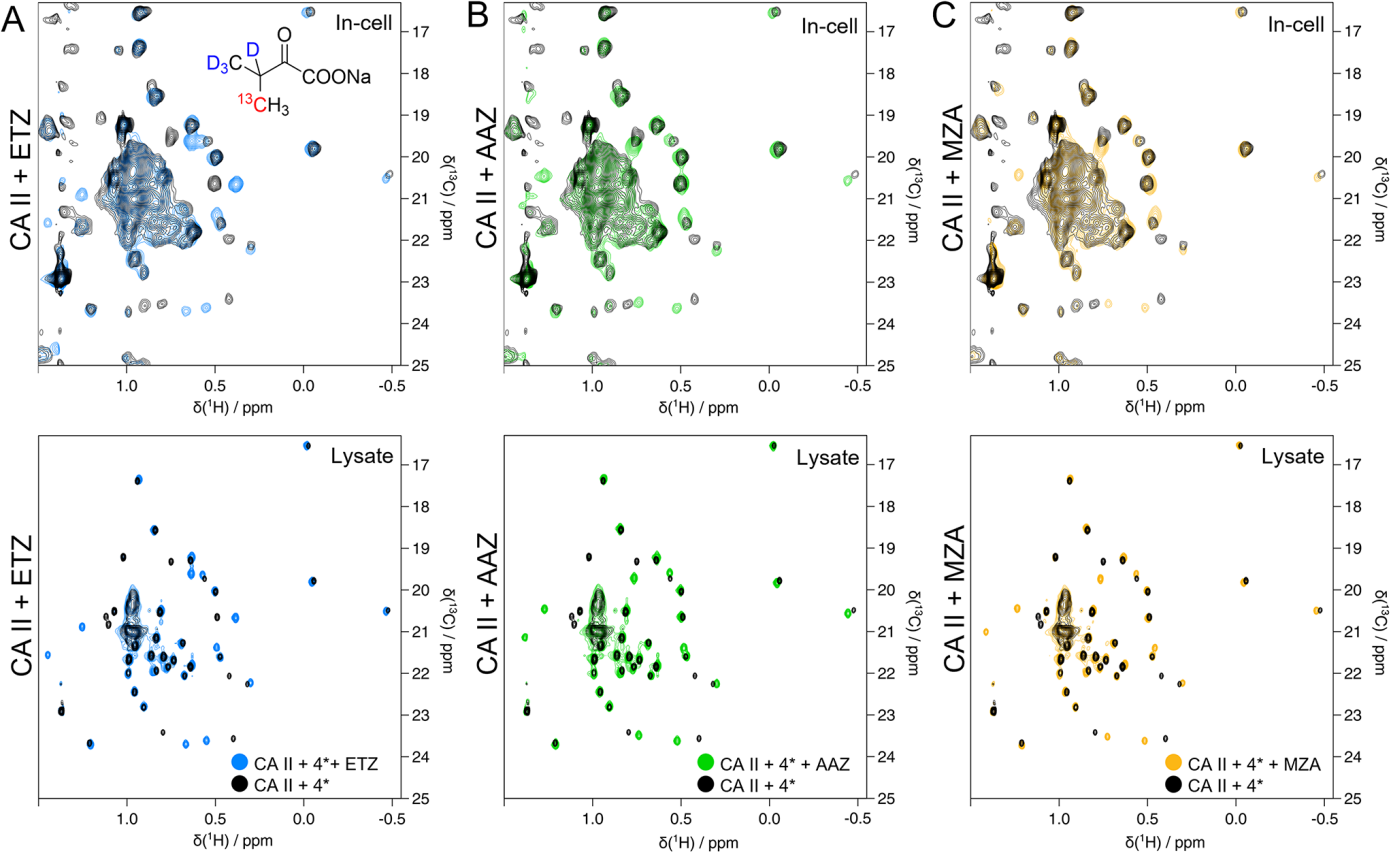
Ligand binding to CA II. (**A**) Top: In-cell NMR spectra on HEK 293T overexpressing CA II, incubated with 4* and treated with ETZ (light blue); In-cell NMR spectra on HEK 293T overexpressing CA II, incubated with 4* (black) Bottom: NMR spectra on HEK293T lysate overexpressing CA II, incubated with 4* and treated with ETZ (light blue); In-solution spectra on HEK293T lysate overexpressing CA II, incubated with 4* (black). (**B**) Top: In-cell NMR spectra on HEK 293T overexpressing CA II, incubated with 4* and treated with AAZ (green); In-cell NMR spectra on HEK 293T overexpressing CA II, incubated with 4* (black). Bottom: NMR spectra on HEK293T lysate overexpressing CA II, incubated with 4* and treated with AAZ (green); In-solution spectra on HEK293T lysate overexpressing CA II, incubated with 4* (black). (**C**) Top: In-cell NMR spectra on HEK 293T overexpressing CA II, incubated with 4* and treated with MZA (yellow); In-cell NMR spectra on HEK 293T overexpressing CA II, incubated with 4* (black). Bottom: NMR spectra on HEK293T lysate overexpressing CA II, incubated with 4* and treated with MZA (yellow); In-solution spectra on HEK293T lysate overexpressing CA II, incubated with 4* (black)

To show an example of a possible application of this labelling technique, we chose a set of well-characterized sulfonamide-derived CA inhibitors: Acetazolamide (AAZ) and methazolamide (MZA) which are currently in use as drugs to treat glaucoma, and ethoxzolamide (ETZ) which is an inhibitor of CAs in proximal renal tubules, widely used as a diuretic. All three compounds were previously shown to tightly bind CA II by in vitro and in-cell NMR (Luchinat et al. 2020a, b). We selected the valine precursor 4* as Val residues are present in the CA II ligand binding site. Indeed, from the spectra comparison it is possible to appreciate the change in the shifts of those valine residues involved in the interaction or experiencing an environment variation due to the proximity of the ligand (Fig. 5).

## Discussion and conclusions

Exclusive isotope labelling of backbone positions is often not sufficient to unfold the full potential of NMR spectroscopy and perform in-depth protein characterization. Special isotope patterns in the side chains are required to investigate interaction surfaces via chemical shift perturbation or study side-chain dynamics in spin relaxation experiments. However, selective isotope labelling is often compromised by high costs for the heavy isotope-containing amino acids needed. Metabolic precursors such as α-ketoacids can be efficiently synthesized from low-cost heavy isotope-containing starting compounds; the corresponding protocols have been summarized in various reviewing articles (Schörghuber et al. 2018; Kerfah et al. 2015; Tugarinov et al. 2006; Rowlinson et al. 2022; Lichtenecker et al. 2015). Many of these precursor isotopologues are even commercially available today. Our results show that using precursors to introduce side chain-labelled amino acids is not limited to *E. coli* expression but can be transferred to mammalian cell hosts. This is especially useful when bacterial or yeast hosts are not viable options to produce a mammalian protein, which is usually the case for large proteins or proteins that undergo post-translational modifications. We furthermore demonstrated that the efficacy of the method is guaranteed even in the case of precursor combinations, granting an amino acid-selective labelling on more than one amino acid, thus expanding the possible applications of this labelling technique. The successful expression of proteins in the presence of the precursors employed in this work is allowed by the presence of transaminases in cultured mammalian cells: the reversible transamination of Ile, Leu, and Val is catalyzed by branched chain amino acid amino transferase (BCAT), Tyr and Phe are substrates of tyrosine aminotransferase (TAT) and glutamic-oxaloacetic transaminase (GOT), the transamination of Met is catalyzed by glutamine transaminase K (GTK), and His is a substrate of both glutamine transaminase L (GTL) and serine-pyruvate transaminase (SPT) (Caligiore et al. 2022; Neinast et al. 2019; Toyokawa et al. 2021; Sivaraman and Kirsch 2006; Mehere et al. 2010; Cooper 2004). Concerning the general applicability of the method, our results suggest that α-ketoacid precursors of other amino acids could be employed, provided that they are recognized by transaminases expressed in the cell culture. GTK and TAT are reported to catalyze the transamination of Trp with high efficiency, whereas a similar activity for Lys and Arg is not reported (Caligiore et al. 2022).

Furthermore, side chain isotope labelling may not be straightforward for some amino acids (Ala, Asp, Asn, Pro, Glu) that are directly connected to core cellular metabolic pathways, such as glycolysis and the TCA cycle. In such cases, adding the corresponding α-ketoacid precursors to the culture medium will likely result in the unwanted labelling of other amino acids in the expressed proteins. Notably, however, the above amino acids are not predominant in protein-ligand and protein-protein interfaces, and therefore their labeling would not be the first choice for studying such phenomena.

As a first example, we demonstrated how this labelling technique results in well-resolved methyl resonances in the NMR spectra of two globular proteins, the 29 kDa CA II and the 40 kDa DJ-1 homodimer. Addition of selected known ligands led to substantial perturbation of the CA II chemical shifts, which could be recorded in-lysate, as well as in-cell. This observation implies that combinations of precursors and mammalian expression systems are generally applicable to investigate protein-ligand- and, in principle, protein-protein interactions. Overall, we believe that introducing α-ketoacid precursors to mammalian cell-based protein expression will greatly facilitate the application of NMR to study structure and function of challenging human proteins in a purified state, in cell lysates, or even in intact cells.

## Electronic supplementary material

Below is the link to the electronic supplementary material.

Supplementary Material 1

## Data Availability

The raw NMR data will be made available upon reasonable request.
