# Supplementary material for: Towards cost-effective side-chain isotope labelling of proteins expressed in human cells
Source: J Biomol NMR. 2024 Aug 22;78(4):237–47. doi: 10.1007/s10858-024-00447-6 (PMC11615012; doi:10.1007/s10858-024-00447-6)
Supplement: Supplementary file 1 — Supplementary Material 1 [file 10858_2024_447_MOESM1_ESM.docx]

**Supporting Information**

**Towards cost-effective side-chain isotope labelling of proteins expressed in human cells**

Martina Rosati^1^, Letizia Barbieri^1,2^, Matus Hlavac^3^, Sarah Kratzwald^3^, Roman J. Lichtenecker^3,4^, Robert Konrat^3,5^, Enrico Luchinat^1,2,6,^*, Lucia Banci^1,2,6.^*

^1^ CERM ─ Magnetic Resonance Center, Università degli Studi di Firenze, Sesto Fiorentino, Italy

^2^ Consorzio Interuniversitario Risonanze Magnetiche di Metallo Proteine ─ CIRMMP, Sesto Fiorentino, Italy.

^3^ MAG-LAB GmbH, Vienna, Austria.

^4^ Institute of Organic Chemistry, University of Vienna, Vienna, Austria.

^5^ Department of Structural and Computational Biology, Max Perutz Laboratories, University of Vienna, Vienna, Austria.

^6^ Dipartimento di Chimica, Università degli Studi di Firenze, Sesto Fiorentino, Italy.

* Enrico Luchinat: eluchinat@cerm.unifi.it; Lucia Banci: banci@cerm.unifi.it

**Supplementary Figures S1-S4**

**Supplementary Figures**


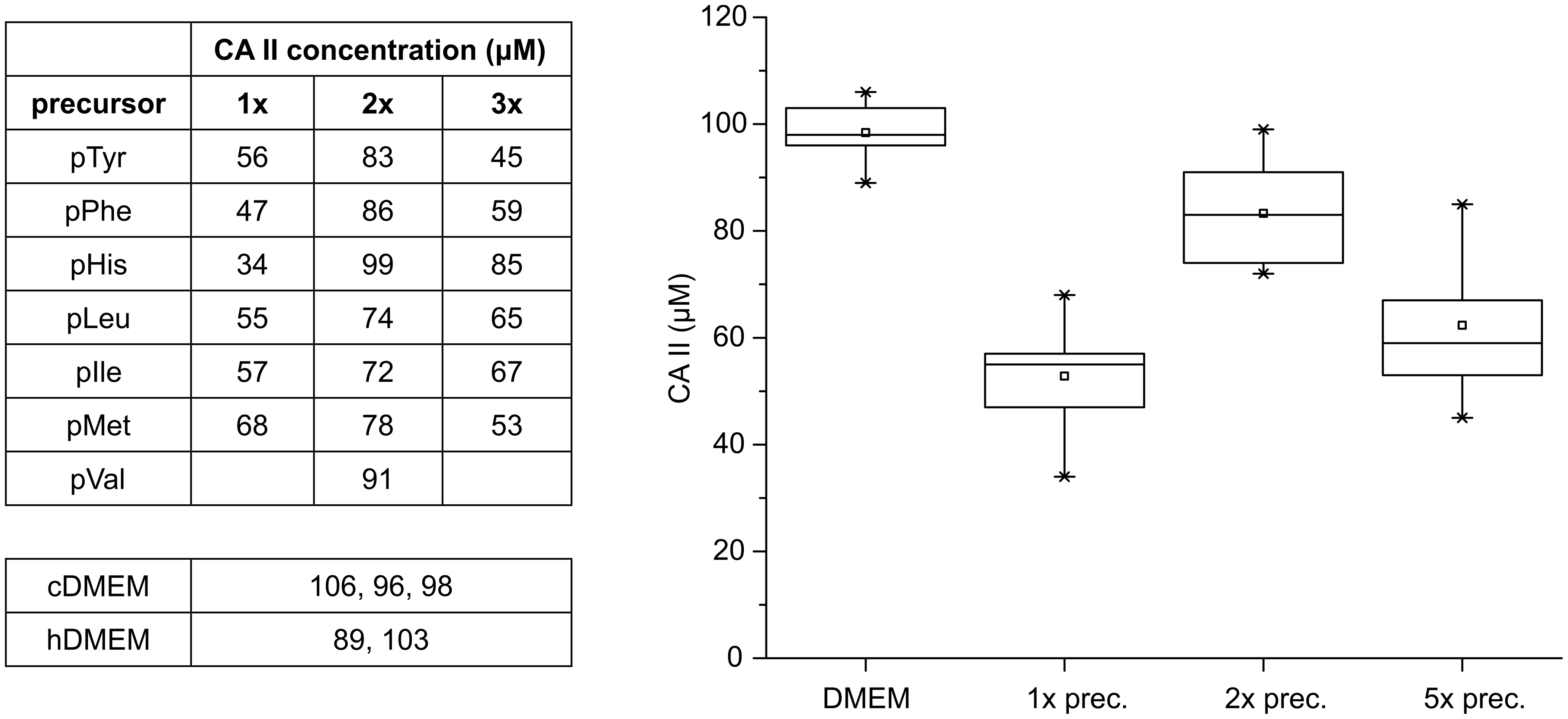


**Fig. S1** Left: CA II concentration in the lysates obtained from cell samples treated with different doses of each precursor. Right: box plot showing the range of CA II concentrations obtained in DMEM (either commercial or custom made) and in custom medium containing 1x, 2x and 5x doses of precursor.


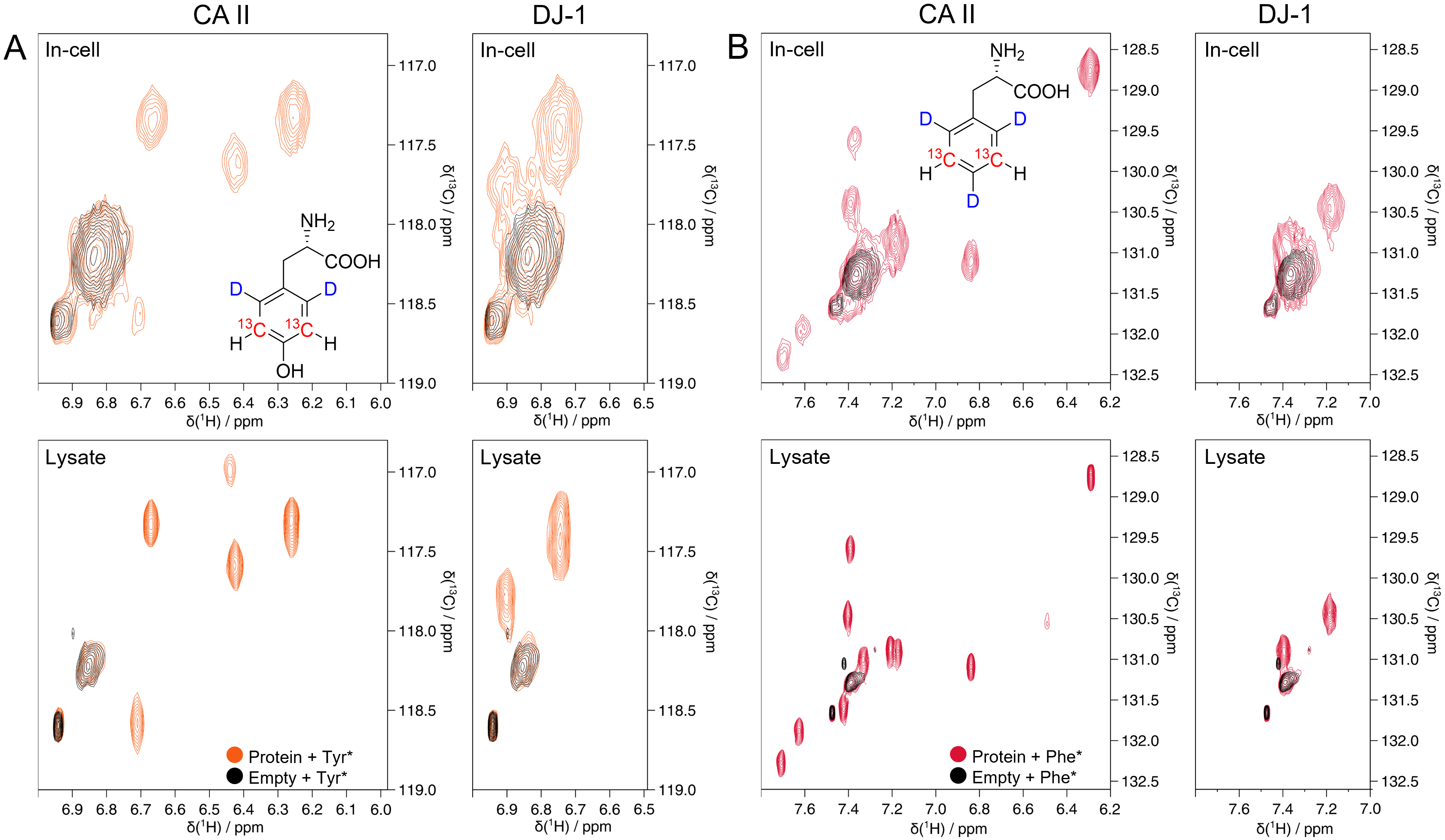


**Fig. S2** Phe* and Tyr* NMR spectra. A) Top: In-cell NMR spectra on HEK293T cells overexpressing CA II (left) and DJ-1 (right), incubated with Tyr* (orange); HEK293T cells transfected with empty vector, incubated with Tyr* (black). Bottom: NMR spectra on HEK293T lysate. Lysate of cells overexpressing CA II (~60 µM, left) and DJ-1 (right), incubated with Tyr* (orange); lysate of cells transfected with empty vector, incubated with Tyr* (black). B) Top: In-cell NMR spectra on HEK293T cells overexpressing CA II (left) and DJ-1 (right), incubated with Phe* (red); HEK293T cells transfected with empty vector, incubated with Phe* (black). Bottom: NMR spectra on HEK293T lysate. Lysate of cells overexpressing CA II (~60 µM, left) and DJ-1 (right), incubated with Phe* (red); lysate of cells transfected with empty vector, incubated with Phe* (black).


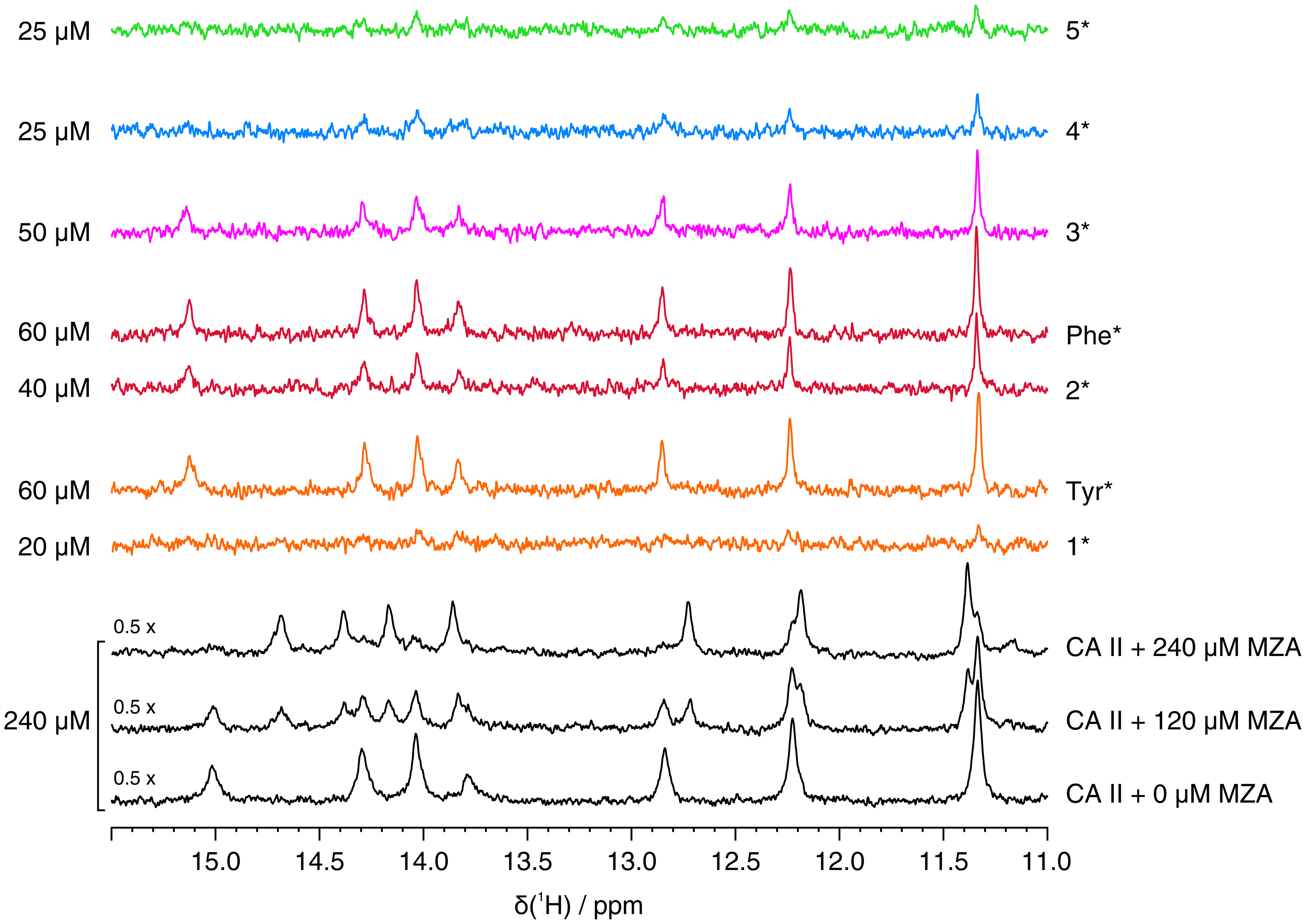


**Fig. S3** CA II quantification in the cell lysates by ^1^H NMR signal integration. From bottom to top: reference cell lysate containing 240 µM CA II (black) titrated with methazolamide (MZA); lysates from cells incubated with **1*** and Tyr* (orange); lysates from cells incubated with **2*** and Phe* (red); lysates from cells incubated with **3*** (magenta), **4*** (blue) and **5*** (green). Estimated CA II concentrations are reported for each sample.

**
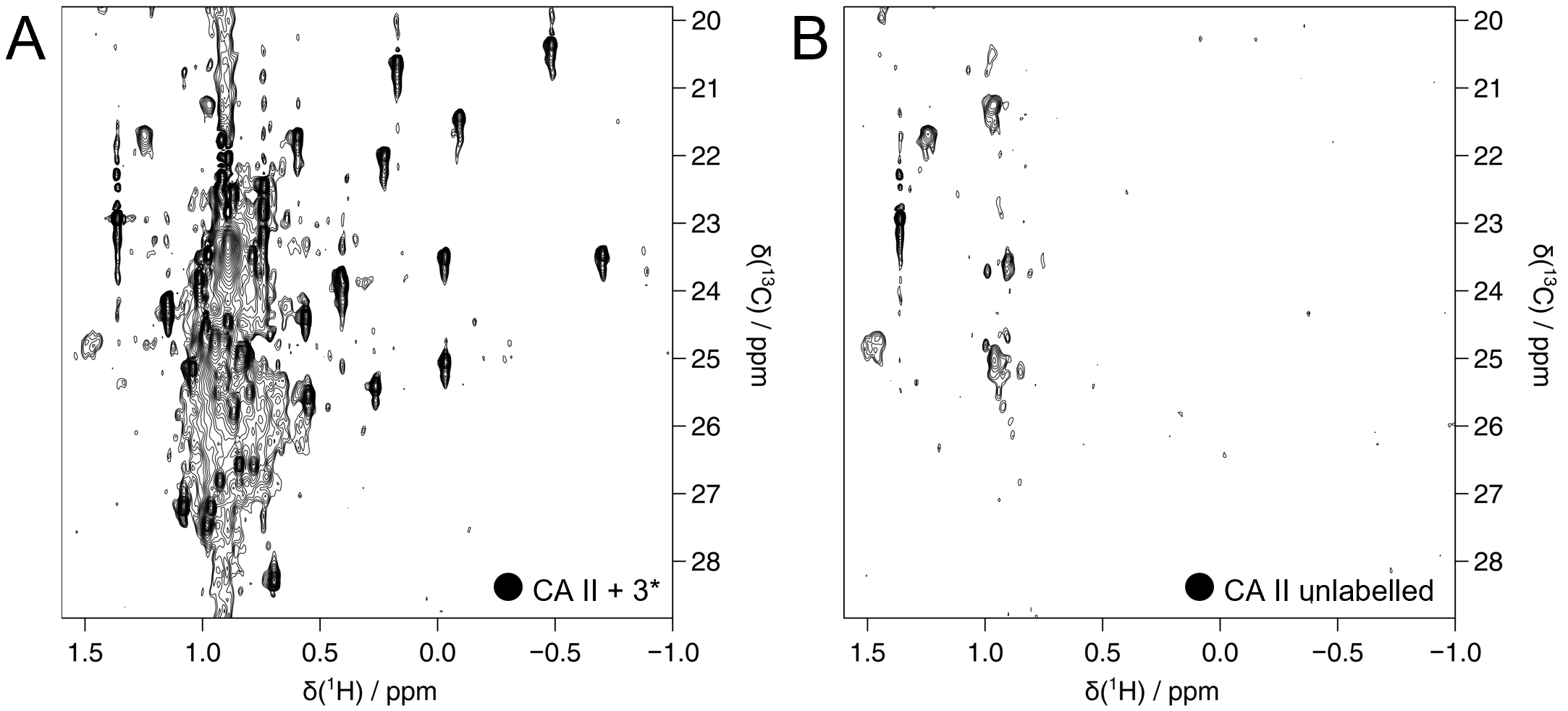
**

**Fig. S4** A) NMR spectrum of a lysate from cells overexpressing leucine-labelled CA II; B) NMR spectrum of a lysate from cells overexpressing unlabelled CA II, recorded with the same duration of (A).
